# Characterization of the Gastrointestinal and Reproductive Tract Microbiota in Fertile and Infertile Pakistani Couples

**DOI:** 10.3390/biology11010040

**Published:** 2021-12-28

**Authors:** Ammara Manzoor, Saira Amir, Farzana Gul, Muhammad Abubakar Sidique, Masood ur Rehman Kayani, Syed Shujaat Ali Zaidi, Sundus Javed, Syed Tahir Abbas Shah, Arshan Nasir

**Affiliations:** 1 Department of Biosciences, COMSATS University Islamabad, Islamabad 45550, Pakistan; emmaramenzor24@gmail.com (A.M.); saira_amir@comsats.edu.pk (S.A.); farzanagul671@gmail.com (F.G.); abubakar32103@gmail.com (M.A.S.); sundus.javed@comsats.edu.pk (S.J.); syedtahirabbas@comsats.edu.pk (S.T.A.S.); 2 Independent Researcher, Los Alamos, NM 85545, USA; masood.kayani@ymail.com; 3 Center for Innovation in Brain Science, Department of Neurology, University of Arizona, Tucson, AZ 85719, USA; syedzaidi@arizona.edu

**Keywords:** gut microbiome, genital microbiome, infertility, 16S ribosomal RNA (rRNA) gene sequencing, α-diversity

## Abstract

**Simple Summary:**

We describe microbial taxa associated with the gastrointestinal and reproductive tracts of married Pakistani couples. We highlight differences in microbial composition and diversity that are associated with fertile and infertile couples and provide a baseline for future in-depth studies to target the association of the human microbiome with infertility.

**Abstract:**

The human microbiota is recognized as a vital “virtual” organ of the human body that influences human health, metabolism, and physiology. While the microbiomes of the gut, oral cavity, and skin have been extensively studied in the literature, relatively little work has been done on characterizing the microbiota of the human reproductive tract organs, and specifically on investigating its association to fertility. Here, we implemented a 16S ribosomal RNA (rRNA) amplicon sequencing approach to sequence and characterize the gut and genital tract microbiomes from several married Pakistani couples. The recruited individuals included 31 fertile and 35 infertile individuals, with ages ranging from 19–45 years. We identified several fluctuations in the diversity and composition of the gut and genital microbiota among fertile and infertile samples. For example, measures of α-diversity varied significantly between the genital samples donated by fertile and infertile men and there was overall greater between-sample variability in genital samples regardless of gender. In terms of taxonomic composition, Actinobacteria, Bacteroidetes, and Firmicutes fluctuated significantly between the gut microbiomes of fertile and infertile samples. Finally, biomarker analyses identified features (genera and molecular functions and pathways) that differed significantly between the fertile and infertile samples and in the past have been associated with bacterial vaginosis. However, we emphasize that 16S amplicon data alone has no bearing on individual health and is merely representative of microbial taxonomic differences that could also arise due to multiple other factors. Our findings, however, represent the first effort to characterize the microbiome associated with fertile and infertile couples in Pakistan and will hopefully pave the way for more comprehensive and broad-scale investigations in the future.

## 1. Introduction

Infertility is typically characterized by a failure to conceive after regular intercourse for twelve or more months [1]. In women older than 35, the timeline to conceive is shortened to six months to improve the odds of successful infertility treatment [2]. Approximately 10–15% of the reproductive-aged couples worldwide may suffer from some kind of infertility during their lifespan [3]. According to various estimates, 20–30% males [4] and ~37% females may experience infertility at some timepoint worldwide [5]. According to the U.S. Centers for Disease Control and Prevention statistics, ~8.8% of married women may not become pregnant within 12 months of trying in the U.S. [6]. The rate of infertility is presumably higher in the developing countries; for instance, infertility prevalence in Pakistan could be as high as 22% for primary infertility accounting for 4% of the total infertility cases [7] suggesting that several married couples in Pakistan may be struggling with infertility [8]. The prevalence of secondary infertility is also presumably higher in Pakistan [9].

Infertility can be caused by genetic, emotional, social, physical (e.g., injury to reproductive organs), and biological/natural factors [10]. Some of the primary factors include declining reproductive age of marital partners and ovulation defects in women and spermatogenic failure and physical injuries in men, among others [11]. In men, infertility can be diagnosed by routine semen analysis, which evaluates sperm morphology, motility, and quantity per ejaculate [12]. For women, several physical and emotional factors may contribute to infertility. These include obstructions in the reproductive tract, endometriosis, polycystic ovarian syndrome (PCOS), pelvic inflammatory disease, hyperprolactinemia, hypothyroidism, and T-shaped uterus [13]. Fortunately, many advanced treatments for infertility are now available worldwide and also in Pakistan. These include ovulation drugs to induce ovulation, intrauterine insemination for artificial insemination (IUI), and in vitro fertilization (IVF), among others, to support embryo fertilization outside the women’s body [14,15]. These treatments can have a legitimate chance of success but their high costs, and social stigma associated with seeking infertility diagnosis and treatment in societies such as Pakistan, may discourage or prevent many infertile couples to seek treatment [16]. 

In recent years, advancements in bioinformatics and (meta)-genomics have targeted the sequencing and description of microorganisms associated with the human body and have confirmed their beneficial roles in host health [17]. Numerous studies have reported the association of dysbiosis or imbalance in the normal human microbiota composition with the initiation and progression of major human diseases such as diabetes, inflammatory bowel disease, cancer, neuropsychiatric diseases, and cardiac metabolic disorders [18]. However, relatively fewer studies have documented the association of microbiome dysbiosis to infertility [19] It is well known that pathogens such as *Neisseria gonorrhoeae* and *Chlamydia trachomatis* can be sexually transmitted and may lead to infertility [20]. These pathogens can cause pelvic inflammatory disease and fallopian tube infection and damage in women [21,22]. Similarly, bacterial vaginosis (BV) is characterized or caused by a change from *Lactobacilli* dominated microbial community to a community dominated by anaerobic bacteria in the vaginal microbiome [23]. BV can lead to an increase in reactive oxygen species (ROS) production, which can cause lipid peroxidation in spermatozoa [24]. A recent study reported a higher *Lactobacillus* count in semen samples from healthy individuals than prostatitis patients [25]. Another study reported similar results showing a higher number of *Lactobacillus* species in the semen of normozoospermic individuals than those with spermatic abnormalities [26]. While *Lactobacillus* concentration has been correlated with normal seminal parameters in healthy individuals [27], *Pseudomonas* and *Prevotella* were related to male infertility in terms of low-quality semen parameters [28]. Moreover, some studies have linked the success of IVF and Intra-cytoplasmic sperm injection (ICSI) with a higher relative abundance of *Lactobacillus* in the vaginal microbiome [29]. Taken together, these studies suggest the role of differential microbiota composition and abundance in fertility outcomes. On similar lines, PCOS and endometriosis have been linked to an altered gut microbiome between control and women diagnosed with PCOS and endometriosis [30,31,32,33]. Specifically, female rhesus monkeys with endometriosis showed decreased levels of *Lactobacilli* and an increase in the concentration of Gram-negative bacteria [34]. However, the mechanisms through which the vaginal and gut microbiota might impact the progression of infertility remain relatively less clear [35].

Infertility may also lead to social problems, especially in developing countries. Infertility may occur due to either male- or female-related factors and sometimes involve both partners [36]. However, historically, and especially in developing countries such as Pakistan, infertility is typically considered a women-specific condition [37]. Pakistani men are traditionally more protective of their masculinity (and ego) and they may even resist infertility diagnosis and treatment [38]. In such societies, infertile couples may face several challenges such as martial conflicts (e.g., fear of separation), treatment-related concerns, sexual dysfunction, personal anxieties (e.g., desire to become parents), and psychosocial and emotional problems resulting from social and family pressures (e.g., depression) [39]. In this study, we, therefore, characterized the gut and reproductive tract microbiome composition/diversity through 16S rRNA sequencing in several married Pakistani couples recruited from various major cities in the country. We observed several fluctuations in the diversity and composition of the gut and genital microbiome in both male and female samples, which we report below, and hope that these findings will provide a baseline for future more specific studies on this topic.

## 2. Materials and Methods

### 2.1. Participant Identification and Screening

Through contacts with local clinics and universities, we initially identified 45 married couples for study enrollment. These couples were divided into 22 fertile and 23 infertile couples, where one or both partners could either be fertile or infertile. Out of these 45 couples, 66 individuals including 32 females (14 fertile, 18 infertile) and 34 males (17 fertile and 17 infertile) who met the study enrollment criteria (read below) were recruited for the study. Initially, all couples actively trying for pregnancy for more than one year with no conception were categorized as infertile. Next, all participants completed an extensive online questionnaire to self-report and identify any medical condition which could lead to infertility such as prior diagnosis with PCOS or endometriosis in women and abnormal semen parameters in men. All couples with kids who had no difficulty in conceiving after the first year of marriage were considered fertile and hence categorized as controls. Out of the 23 infertile couples, 7 had fertility problems involving both partners (30%), 10 involved only male factor infertility (43%), 3 involved female factor infertility (13%), while no apparent cause or reason of infertility could be established in the remaining 3 couples (13%). In cases where one of the partners was unavailable for sampling, only one partner was sampled. The study exclusion criteria included either the use of antibiotics or travel abroad in the last three months, women on menopause, and participants suffering from inflammatory bowel disease [40]. Both male (mean age 33.97 ± 6.14) and female (mean age 28.25 ± 5.47) participants belonged to the reproductive age (i.e., 19–45) years. All participants provided written informed consent to participate in the study. This study was approved by the Ethics Review Board of COMSATS University Islamabad (CUI) (protocol number: CUI/Bio/ERB/03-19/23 approved on 25 March 2019). 

### 2.2. Sample Collection

Each recruited study participant was instructed to provide two biological samples: (i) fecal sample, which was treated as the proxy for the gut microbiome, and (ii) the genital sample. Samples were self-collected by the study participants following the uBiome protocol for sampling (as also described in our previous study [40]). Participants were briefed about sampling instructions both in writing and in their native language. Specifically, participants were instructed to use a sterile swab soaked in PCR water, provided in the uBiome sampling kit, and then asked to swirl the swab around the genitals for at least one minute. After sampling, swabs were mixed in the DNA lysis and stabilization buffer, provided with the uBiome kits. For fecal samples, sterile wipes and swabs were provided in the uBiome kit. Participants were instructed to wash their hands prior to sampling and to collect the first bowel movement early in the morning while fasting. A small amount of feces from the wipe was then transferred to the sterile swab, which was mixed in the stabilization buffer for one minute, similar to the genital sampling protocol. Samples were stored at room temperature at CUI prior to shipping to uBiome, USA, for subsequent steps. 

### 2.3. 16S Ribosomal RNA (rRNA) Gene Sequencing, DNA Extraction, and PCR Amplification

DNA extraction, PCR amplification, and sequencing were performed by the uBiome laboratory in California. In brief, samples were lysed via mechanical bead beating [41]. DNA extraction followed the protocol of [42]. The V4 region of the 16S ribosomal RNA (rRNA) gene was amplified using universal forward and reverse primers. Illumina barcodes and sequence tags were added to primers for multiplexing. PCR products were selected based on size following the protocol in Minalla et al. (2001) [43]. Multiplexed 150 bp paired-end data was generated for 16S amplicons using the NextSeq 500 platform. 

### 2.4. Quality-Control

Quality-controlled reads provided by uBiome were analyzed locally using QIIME2 [44]. A quick check on quality indicated that reverse reads were, on average, of lower quality than the forward reads. We, therefore, performed bioinformatics analysis only on the forward reads. Pre-processed forward reads were imported into QIIME2 using the ‘import’ plugin and were capped to 125 bp. Additional chimera removal, denoising, and Amplicon Sequence Variants (ASVs) were produced by the DADA2 plugin in QIIME2. Alignment of representative sequences was performed using MAFFT [45], which was subsequently used to generate the unrooted and rooted phylogenetic trees of representative sequences using FastTree [46]. Taxonomic classification was performed using SILVA 128 database [47].

### 2.5. Evaluation of within and between Sample Diversity

Samples were rarefied to a depth of 5000 prior to diversity analysis. Rarefaction removed one genital sample with a very low read count (see below). Standard phylogenetic and non-phylogenetic α-diversity indicators such as the observed number of ASV’s per sample [48], Faith’s phylogenetic diversity (PD) [49], Shannon’s diversity index [50], and Pielou’s evenness were used to evaluate within-sample diversity [51]. Similarly, standard β-diversity indicators such as the Bray–Curtis dissimilarity [52], Jaccard Distance [53], weighted UniFrac [54] and unweighted UniFrac [55] were calculated on all samples. The α-group significance was evaluated by the non-parametric Kruskal–Wallis (KW) test. The β-group significance was evaluated using PERMANOVA with 999 permutations. Principal coordinate analysis (PCoA) was visualized using Emperor [56] to evaluate sample dissimilarity at key metadata variables.

### 2.6. Biomarker Discovery

The linear discriminant analysis (LDA) effect size (LEfSe) [57] method from the Galaxy online server (https://huttenhower.sph.harvard.edu/galaxy/) (accessed on 15 December 2019) was used to identify features that were differentially abundant in classes (fertile and infertile) and subclasses (male and female). The LDA threshold was relaxed from 2.0 (default) to 3.0 for these comparisons.

### 2.7. Metagenome Function Prediction

The functional metagenome of samples was predicted by the PICRUST2 plugin [58] for QIIME2. The three predicted metagenomes were the (i) EC metagenome representing the abundance of features classified by enzyme commission numbers [59], (ii) KO metagenome indicating KEGG orthologs abundance [60], and (iii) the MetaCyc pathways abundance [61]. For all three functional annotations, standard non-phylogenetic α and β diversity indicators were calculated on all samples. Significantly abundant bacterial taxa among fertile and infertile subjects for both body sites and genders were detected by the LEfSe method from Galaxy online server, as above.

## 3. Results

We sequenced the 16S rRNA (V4 region) for a total of 107 samples donated by 66 selected participants. For some individuals, both gut and genital samples were available and for the rest, only one body site was sequenced. In total, 107 samples included 48 gut and 59 genital samples. The mean number of detected features was 94,067 per sample, ranging from 977 to 524,730. The second smallest sample had 7236 features. Therefore, we rarefied the feature table to a sampling depth of 5000 features per sample and removed one genital sample with the smallest count. Subsequent analysis was therefore done on 106 retained samples (48 gut and 58 genital), each with 5000 features.

### 3.1. Microbial Taxonomic Composition of Body Sites in Fertile and Infertile Samples

**Figure 1:**
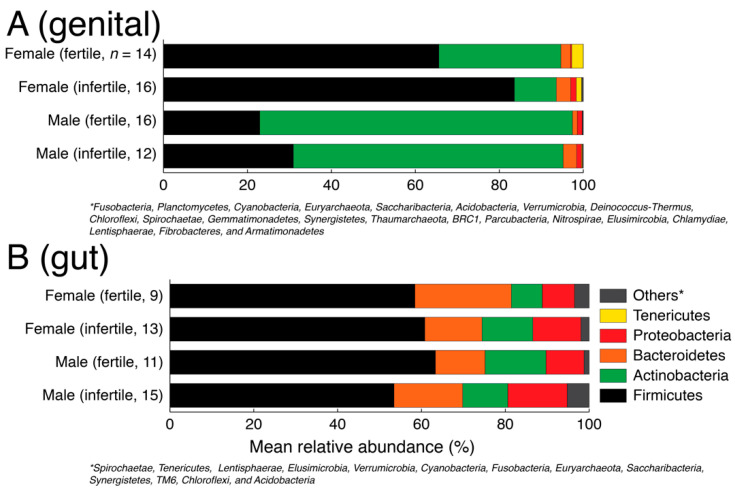
Phylum level taxonomic composition of genital (**A**) and gut (**B**) samples stratified by gender and fertility status.

First, we evaluated the phylum-level taxonomic composition in the genital and gut samples for fertile and infertile men and women (Figure 1). For this analysis, we partitioned the rarefied feature table into the gut and genital samples and transformed raw abundance counts into relative abundance values. A total of 26 phyla (including one unassigned) were detected in the genital samples and 18 phyla (one unassigned) were detected in gut samples. Some of these phyla were detected in a very small number of samples. In both gut and genital samples, five major phyla dominated the microbial communities. These included Firmicutes, Actinobacteria, Bacteroidetes, Proteobacteria, and Actinobacteria. The rest were pooled into the ‘others’ category (Figure 1). The gut samples were dominated by Firmicutes and Bacteroidetes (Figure 1 and Table 1). The ratio or imbalance between these two key phyla has previously been linked to obesity [62]. Though, some authors have questioned the claim [63]. In our gut samples, Firmicutes dominated Bacteroidetes (mean relative abundance ranging from 53–63% vs. 11–23%, Table 1), which could indicate a tendency towards weight gain [40]. Firmicutes were significantly more abundant in fertile men vs. infertile men (63.32% vs. 53.45%, *p* = 0.03, two-tailed Mann–Whitney test) while Bacteroidetes were significantly more abundant in fertile women vs. infertile women (23.01% vs. 13.64%, *p* = 0.025, two-tailed Mann–Whitney test). In turn, Actinobacteria were significantly more abundant in infertile women vs. fertile women (12.12% vs. 7.36%, *p* = 0.036, two-tailed Mann–Whitney test). Since the gut microbiome is strongly influenced by diet and we did not have a sufficiently larger sample size, we caution the readers to interpret these numerical differences with caution.

In turn, there was a relatively greater numerical imbalance in the taxonomic composition between genders and fertility status in the genital samples (Figure 1) but no statistically supported differences (Table 1). For example, Firmicutes were numerically over-represented in infertile women vs. fertile women (83.57% vs. 65.58% mean relative abundance) and Actinobacteria were numerically under-represented in infertile women vs. fertile women (10% vs. 29% mean relative abundance) but the distribution differences were statistically insignificant (*p* > 0.05, two-tailed Mann–Whitney test) possibly indicating greater individual-to-individual variability. Similarly, Proteobacteria were numerically over-represented in infertile women vs. fertile women (1.32% vs. 0.27%), which has previously been linked with BV [64] and preterm birth [65] but again the differences were statistically insignificant (*p* = 0.95, two-tailed Mann–Whitney test). This is likely because of the many factors behind infertility and a smaller sample size for each specific factor analyzed in this study.

To zoom into these differences, we next identified the top five (5) most abundant genera in each unique combination of the body site, gender, and fertility status (Table 2). In infertile women, the genital microbiome revealed a relative increase in the populations of *Lactobacillus*, *Atopobium*, and *Prevotella* (Table 2). *Lactobacillus*, in general, is associated with a healthy pregnancy and is considered a beneficial microorganism in the women reproductive tract [66]. Therefore, our observation of a higher relative increase in *Lactobacillus* abundance in infertile women merits further investigation. However, relative increases in the abundances of *Atopobium* and *Prevotella* in the vaginal microbiome of infertile women were also reported in a recent study to characterize the vaginal microbiome in women experiencing secondary infertility [19]. The authors concluded that a combination effect caused by higher abundances of these genera, among others, was probably a contributing factor in infertility [19]. The gut microbiome also revealed fluctuations in key genera populations between fertile and infertile men and women. A notable appearance was of *Succinivibrio* population in infertile men (8.23% of total community), which are potentially sugar-metabolizing bacteria [67] and were previously detected in higher amounts in Pakistani men [40]. In turn, *Succinivibrio* was not amongst the top five genera in the gut microbiome of fertile men (Table 2).

Taken together, various fluctuations in the relative abundances of key microbial taxa were observed between fertile and infertile individuals for both body sites, along with evidence of significant heterogeneity among individuals. However, given the small sampling sizes and the individual-level heterogeneity, these differences should be interpreted with caution.

### 3.2. Significantly Different within-Sample Diversity in the Genital Microbiome of Fertile and Infertile Men

**Figure 2:**
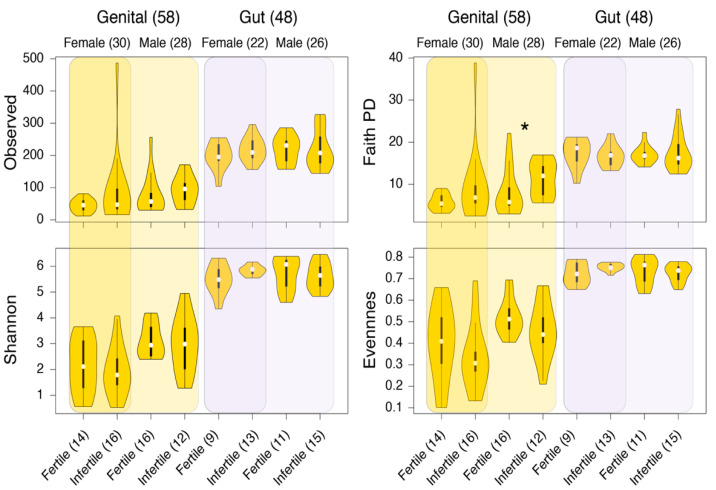
Comparisons of alpha-diversity distributions between the genital and gut samples. Plots are stratified by gender and fertility status. Numbers in parenthesis indicate the total number of samples in that group. * *p* < 0.05, KW test.

The α-diversity of the retained samples was evaluated by four standard measures: (i) the observed ASVs (the number of unique or distinct ASVs in a sample) [48], (ii) Shannon’s diversity index (considers both ASV abundance and evenness in samples) [50], Faith’s PD, which evaluates diversity based on phylogenetic trees [49], and (iv) Pielou’s evenness, which measures the relative evenness of ASVs in samples [51]. Gut samples, on average, indicated higher within-sample diversity compared to genital samples, while genital samples again indicated greater variability (Figure 2). When partitioned by gender and fertility status, only Faith’s PD was significantly different in the genital samples donated by fertile and infertile males (Figure 2). This result is further evident in Table 3, which lists the *p*- and *Q*-values (FDR adjusted) for each comparison, based on the non-parametric pairwise KW test. In general, *p*- and *Q*-values were consistently lower for male genital sample comparisons based on Faith’s PD and Pielou’s evenness. Another marginally significant result was differences in the Shannon’s diversity index for fertile and infertile gut samples donated by sampled women (Table 3). The violin plot distributions in Figure 2 confirm that Shannon’s diversity values for gut samples were relatively more widespread among fertile women than infertile women.

### 3.3. No Visible Structure but Significant between Sample Diversity Differences among Fertile and Infertile Genital Samples

**Figure 3:**
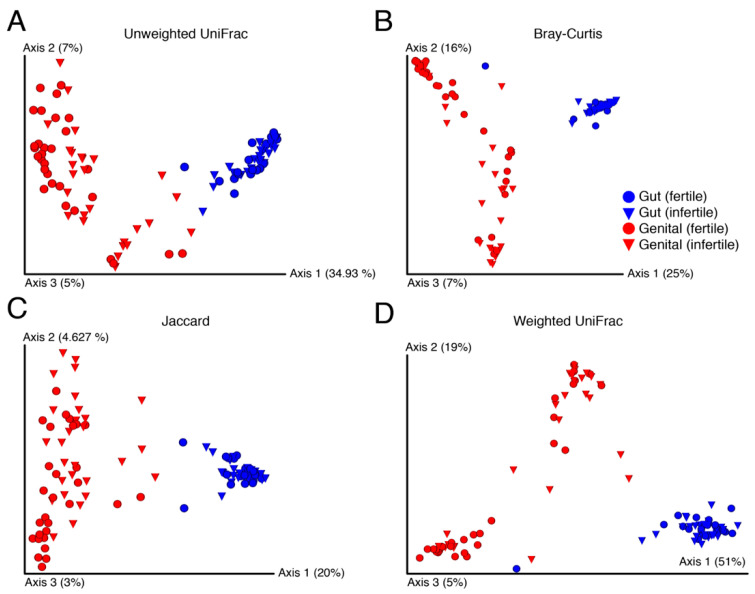
Principal coordinates highlight dissimilarity between samples evaluated by standard beta-diversity indicators ((**A**–**D**), Unweighted UniFrac, Bray-Curtis, Jaccard, and Weighted UniFrace, respectively). Numbers in parentheses indicate percentage variability (%) explained by each axis.

In terms of β-diversity, no visible structure was evident between fertile and infertile samples for any of the four studied measures (Figure 3). These measures included the non-phylogenetic indicators of β-diversity such as the Bray–Curtis and Jaccard and the phylogenetic measures of weighted and unweighted UniFrac. Bray–Curtis quantifies compositional dissimilarity between two different samples [52] while Jaccard measures how dissimilar the two groups are [53]. In turn, the weighed [54] and unweighted UniFrac [55] are derived from phylogenetic trees and calculate phylogenetic distances between pairs of samples. Regardless of the choice of β-diversity indicator, samples clustered by body site rather than by fertility status, which was expected (Figure 3).

In general, gut samples had lower between sample variability than genital samples, which were widespread on the three principal coordinates (Figure 3). This indicated that gut samples from an individual to another were relatively more similar in diversity and independent of the fertility status and gender. In turn, genital samples indicated massive variability from an individual to another but apparently no visible structure by fertility. This likely occurred because of the many varied causes of infertility that may influence each individual differently. Surprisingly, however, fertile genital samples were also equally widespread on the axes. We, therefore, evaluated the significance of β-diversity differences between samples using the PERMAONVA test with 999 permutations. Interestingly, genital β-diversity between fertile and infertile groups was significantly different for both men and women under the relaxed thresholds (Table 4). In comparison, there were no significant differences in gut β-diversity between fertile and infertile men and women, except for the weighted UniFrac measure calculated for females (Table 4).

### 3.4. Biomarker Discovery

**Figure 4:**
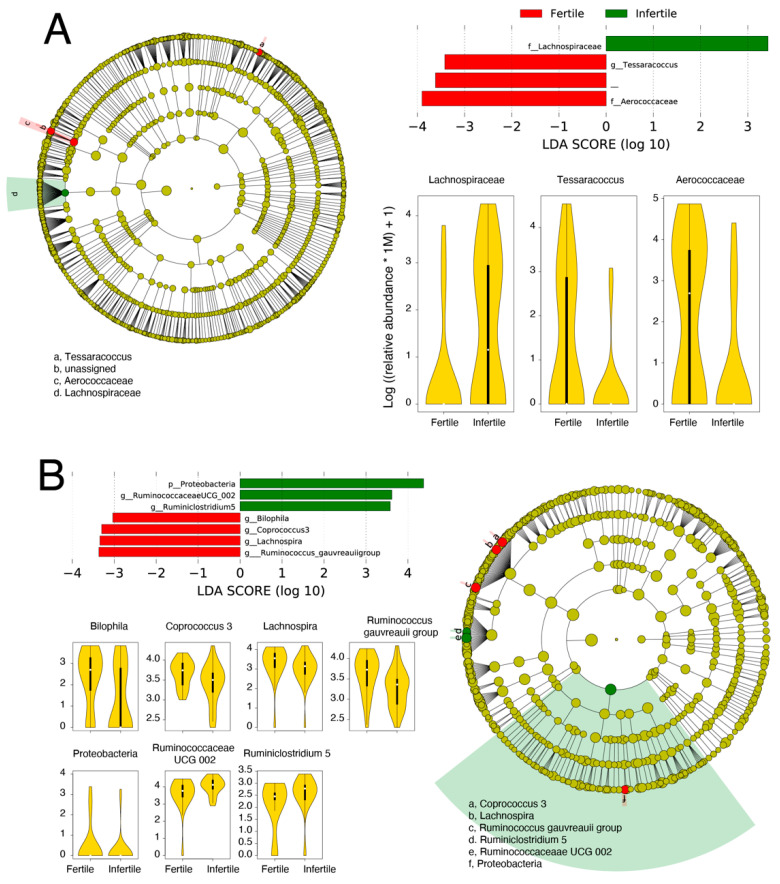
Biomarker discovery in the genital (**A**) and gut (**B**) samples. Microbial taxa differentially abundant between fertile and infertile genital and gut samples are named along with their LDA scores, cladograms, and relative abundance distributions.

Next, we identified key microbial families and genera that were differentially abundant in the genital and gut samples. Differential abundance was evaluated across class (fertility/infertility) and sub-class (female/male) separately for both body sites using LEfSe [57]. For genital samples, members of family *Lachnospiraceae* were significantly more abundant in infertile samples relative to fertile samples (Figure 4A). *Lachnospiraceae sp.* have previously been linked to BV [68]. In turn, the fertile group had significantly higher abundances of family *Aerococcaceae* and genus *Tessaracoccus*. *Aerococcaceae* are a family of Gram-positive lactic acid bacteria and its high relative abundance was detected in women having a high risk of human papillomavirus infection [69]. In turn, *Tessaracoccus* are Gram-positive bacteria that belong to family *Propionibacteriaceae*. In a recent study, *Tessaracoccus* were detected in the vaginal swab of healthy women [70]. It is usually detected in the gut of children suffering from Kwashiorkor (a severe form of malnutrition) [71]. In gut samples, members of phylum Proteobacteria, genus *Ruminococcaceae UCG 002*, and *Ruminiclostridium 5* were significantly more abundant in infertile samples, while genera *Coprococcus 3*, *Bilophila*, *Ruminococcus gauvreauii* group, and *Lachnospira* were significantly overrepresented in fertile samples groups (Figure 4B). Proteobacteria are Gram-negative bacteria that include a notable and wide range of pathogenic genera, for example, *Escherichia*, *Vibrio*, and *Helicobacter*, among several others [72]. The higher abundance of Proteobacteria is generally a marker of dysbiosis in the gut microbiota and potentially a predictor for metabolic diseases [73]. In turn, family *Ruminococcaceae UCG 002* belongs to *Ruminococcaceae*. This genus is usually enriched in the urine of bladder cancer patients [74]. *Ruminiclostridium 5* is a member of family *Ruminococcaceae*. It is significantly more abundant in carbohydrate-utilizing digesta-associated bacterial communities [75]. *Coprococcus 3* is a member of family *Lachnospiraceae*. It is significantly abundant in the gut microbiota of healthy individuals [76]. It is also observed that gout patients have a low relative abundance of genus *Coprococcus 3* [77]. Genus *Bilophila* is an anaerobic, gram-negative Proteobacteria. In human gut microbiota, its significant depletion is detected following insulin intake [78]. Its decreased relative abundance is also observed in autistic individuals [79]. *Ruminococcus gauvreauii* group is an anaerobic Gram-positive bacterium, first isolated from a human fecal specimen and produce acetic acid as a result of fermentation of glucose [80]. Finally, genus *Lachnospira* belongs to family *Lachnospiraceae* and its low relative abundance is detected in alcoholic individuals [81]. Its decreased abundance can also enhance the risk of developing asthma in children [82]. Although, we lack clarification on how these abundances may be associated with infertility.

### 3.5. Metagenome Profiling of Genital and Gut Samples

To visualize the functional repertoire of microbial communities in our studied groups, we predicted the functional metagenome of our samples using PICRUSt2 [58]. We performed functional annotations using KEGG [60], EC [59], and MetaCyc Pathways [61] on the rarefied tables generated previously. The output feature tables were subsequently rarefied to the lowest count for each of the three functions and tested for significant differences using the KW test. Both gut and genital samples were partitioned by gender and fertility status. For the EC metagenome, Pielou’s evenness differed significantly in the genital microbiome of fertile and infertile men (Table 5). Similarly, the gut microbiome differed significantly between fertile and infertile women based on EC annotations (Table 5). Similar results were obtained largely for KEGG (Table 6) and pathway annotations (Table 7). In terms of between sample metagenome variability and beta-diversity, statistically significant differences were detected in different combinations (Table 8, Table 9 and Table 10). To resolve these findings, we performed biomarker discovery on all three annotations and two body sites (Table 11). In total, 14 enzymes including 4 transferases and 3 oxidoreductases, which are known to affect steroid production [83], and 1 KEGG ortholog (Glycerol-3-phosphate dehydrogenase), which is required for sperm motility [84] differed significantly between the fertile and infertile genital samples.

## 4. Discussion

Infertility affects millions of men and women worldwide [3]. It is often associated with stigma and social pressure, especially in developing countries such as Pakistan, where men typically free themselves from any responsibility. While technological advancements such as the wide acceptance of IVF, IUI, and ICSI are now commonly employed across the world to treat infertility, seeking such treatment options in Pakistan may not be as accessible or straightforward.

There are numerous reasons behind the low acceptance of infertility as a treatable disease in Pakistan. First, treatments such as IVF are considered expensive and uncertain. This is largely a consequence of limited awareness as there are several IVF clinics now operating in Pakistan with costs ranging from USD 2500 to USD 4000, which are a fraction of the total IVF-associated costs in developed countries such as the US and in Europe (can be up to USD 40,000 or more) [85]. Whereas IUI may only cost around USD 300–500 per round in Pakistan and is available across most local fertility clinics. These clinics are therefore the choice for several Expat Pakistani couples struggling with infertility and finances [86]. Second, infertility is commonly advertised as a ‘women-only’ problem both in popular Pakistani media and among family circles [87]. Consequently, men even deny diagnosis and women solely suffer the emotional toll of infertility, sometimes leading to separation and divorce. This is unfortunate as our survey shows that 43% of the infertile couples had male-factor infertility. Through this manuscript, we, therefore, wish to emphasize that infertility can affect both men and women and there is no stigma in receiving diagnosis and treatment, especially when they are accessible at a low cost compared to similar options in the most developed countries.

In the recent past, several studies have explored alternative associations that may explain fluctuations in human health and behavior. These include studying the microbial taxonomic composition and phylogenetic diversity associated with the digestive tract to explain various metabolic and physiological diseases [88]. Work has also expanded to the sequencing of the microbiome of the reproductive tract to hopefully understand how microorganisms may contribute towards the reproductive health of the individual [89]. In this study, we, therefore, identified several Pakistani couples struggling with infertility and sequenced their microbiomes from the digestive and reproductive tracts. For comparison, we also sequenced the same microbiomes from fertile controls. To our knowledge, this is the first study of its kind to explore both between and body site microbiome variability among fertile and infertile couples in Pakistan.

Our investigations revealed some strong and some weak statistical differences in the taxonomic structure and diversity of microbial communities associated with the fertile and infertile samples. For example, Actinobacteria and Proteobacteria indicated high fluctuations between fertile and infertile communities sampled from the genitals. Proteobacteria were also relatively more abundant in infertile men who donated stool samples. Proteobacteria is a large phylum of bacteria that includes many notable human pathogens. They are usually a minor part of the gut microbial communities, and a high abundance of *Proteobacteria* in the gut of infertile males indicates gut microbiota dysbiosis, as it is a marker of microbial imbalance in the gut and a potential microbial signature in many disorders [90]. Similarly, the relative abundance of *Prevotella* increased in the genital samples from infertile male and female participants (Table 2). An increase in *Prevotella* has been linked with the failure of assisted reproductive technologies (ART), including IVF and ICSI, resulting in hindrance in conception [65]. Similarly, we also observed a shift in the vaginal microbiome to a community that is associated with BV. The biomarker analyses also revealed similar patterns. For example, the genital samples of infertile persons were enriched with *Lachnospiraceae* (Figure 4) that is strongly linked with BV [91].

Our study has some limitations that need to be addressed in the follow-up studies. The gut microbiome of individuals can vary because of numerous dietary and social habits. For example, a shift from a meat-based to a plant-based diet can alter microbial community composition [92]. Thus, fluctuations in the gut microbiome may neither be the cause nor effect of infertility. Similarly, the level of social stress and the metabolic health of individuals also needs to be properly investigated. Several conditions such as allergies [93], viral and bacterial infections [94], and the past history of vaccination [95] and immunization [96] can also impact the gut microbiome structure and taxonomic diversity. These factors can be tested via thorough medical examinations but in our work have been taken at face value via participant self-reported data. Similarly, infertility can be caused by various reasons such as PCOS, physical injuries, emotional stress, among others [97]. In our work, we pooled all contributing factors under one umbrella. This kind of analysis, therefore, lacks resolution but provides a “bird’s eye” view of the differences in the microbial communities between fertile and infertile samples, which may be due to one or several underlying factors. Further, we relaxed the *p*- and *Q*- thresholds to 0.1 to be able to detect finer differences among groups and subjects. Nevertheless, and to the best of our knowledge, the present work is the first effort to link the microbiome to infertility across Pakistan. We included individuals from diverse ethnicities in the present work and sampled two body sites, where possible. The analysis identified some key biomarker microbial taxa that are significantly and differentially populated between fertile and infertile samples. Their pathology and microbiology need to be better investigated. Similarly, the overall diversity analysis points to structural changes in microbial communities across fertile and infertile samples that also need to be better investigated.

## Figures and Tables

**Table 1:** Mean relative abundance (%) of top five most abundant phyla in each of the eight unique possible combinations of fertility status, body site, and gender.

Body Site	Gender	Fertile		Infertile	
		Phylum	Mean	Phylum	Mean (%)
Genital	Female	Firmicutes	65.58	Firmicutes	83.58
		Actinobacteria	29.07	Actinobacteria	10.01
		Bacteriodetes	2.31	Bacteriodetes	3.42
		Proteobacteria	0.27	Proteobacteria	1.32
		Tenericutes	2.73	Tenericutes	1.27
		Others	0.02	Others	0.41
	Male	Firmicutes	22.92	Firmicutes	30.92
		Actinobacteria	74.49	Actinobacteria	64.27
		Bacteriodetes	1.16	Bacteriodetes	3.18
		Proteobacteria	1.16	Proteobacteria	1.18
		Tenericutes	0.01	Tenericutes	0.20
		Others	0.25	Others	0.23
Gut	Female	Firmicutes	58.43	Firmicutes	60.79
		Actinobacteria	7.36	Actinobacteria	12.12
		Bacteriodetes	23.01	Bacteriodetes	13.64
		Proteobacteria	7.68	Proteobacteria	11.45
		Others	3.51	Others	1.98
	Male	Firmicutes	63.32	Firmicutes	53.45
		Actinobacteria	14.55	Actinobacteria	10.79
		Bacteriodetes	11.84	Bacteriodetes	16.36
		Proteobacteria	9.05	Proteobacteria	14.13
		Others	1.24	Others	5.26

**Table 2:** Mean relative abundance of the top five most abundant genera in each of the eight unique possible combinations of fertility status, body site, and gender.

Body Site	Gender	Fertile		Infertile	
		Genus	Abundance (%)	Genus	Abundance (%)
Genital	Female	Lactobacillus	46.99	Lactobacillus	75.97
		Corynebacterium 1	15.57	Gardnerella	6.33
		Gardnerella	7.58	Atopobium	1.90
		Anaerococcus	4.15	Prevotella	1.85
		Staphylococcus	3.08	Staphylococcus	1.31
	Male	Corynebacterium 1	62.68	Corynebacterium 1	52.19
		Staphylococcus	9.21	Lactobacillus	11.02
		Corynebacterium	8.18	Staphylococcus	8.64
		Anaerococcus	4.78	Corynebacterium	5.96
		Finegoldia	2.82	Prevotella	2.67
Gut	Female	Alloprevotella	11.40	Bifidobacterium	9.47
		Faecalibacterium	6.66	Faecalibacterium	8.04
		f__Lachnospiraceae;__	6.09	f__Lachnospiraceae;	5.89
		Bifidobacterium	5.81	Alloprevotella	4.85
		Bacteroides	5.09	Megasphaera	3.93
	Male	Bifidobacterium	6.27	Succinivibrio	8.23
		Megasphaera	5.63	Bifidobacterium	8.03
		f__Lachnospiraceae;__	5.29	Alloprevotella	6.72
		Faecalibacterium	5.20	f__Lachnospiraceae;	5.92
		Alloprevotella	5.13	Faecalibacterium	5.41

**Table 3:** Alpha-diversity differences in the studied groups. These differences were based on the non-parametric pairwise KW test. *p*- and *Q*-values < 0.1 are in bold (relaxed thresholds to detect more differences).

Method	Group 1	Group 2	*H*	*p*-Value	*Q*-Value
Genital, female					
Observed	Fertile (14)	Infertile (16)	0.6576	0.4174	0.5313
Faith PD	Fertile (14)	Infertile (16)	2.3658	0.1240	0.1736
Shannon	Fertile (14)	Infertile (16)	0.3387	0.5606	0.6561
Evenness	Fertile (14)	Infertile (16)	1.4533	0.2280	0.2776
Genital, male					
Observed	Fertile (16)	Infertile (12)	1.6915	0.1934	0.2708
Faith PD	Fertile (16)	Infertile (12)	4.9655	**0.0259**	**0.0402**
Shannon	Fertile (16)	Infertile (12)	0.1056	0.7452	0.7728
Evenness	Fertile (16)	Infertile (12)	2.7931	**0.0947**	0.1262
Gut, female					
Observed	Fertile (9)	Infertile (13)	1.2154	0.2703	0.3603
Faith PD	Fertile (9)	Infertile (13)	0.6968	0.4039	0.5140
Shannon	Fertile (9)	Infertile (13)	3.6221	**0.0570**	**0.0798**
Evenness	Fertile (9)	Infertile (13)	1.5262	0.2167	0.2758
Gut, male					
Observed	Fertile (11)	Infertile (15)	0.1139	0.7358	0.7924
Faith PD	Fertile (11)	Infertile (15)	0.3562	0.5506	0.6703
Shannon	Fertile (11)	Infertile (15)	0.2970	0.5858	0.6561
Evenness	Fertile (11)	Infertile (15)	0.5663	0.4517	0.5059

**Table 4:** Differences in the beta-diversity among samples, as evaluated by pairwise PERMONVA with 999 permutations. (<0.1 are in bold).

Method	Group 1	Group 2	Sample Size	Permutations	Pseudo-F	*p*-Value	*Q*-Value
Genital, female							
Unweighted UniFrac	Fertile	Infertile	30 (14, 16)	999	1.4235	**0.0730**	**0.0929**
Weighted UniFrac	Fertile	Infertile	30 (14, 16)	999	3.2966	**0.0240**	**0.0320**
Bray–Curtis	Fertile	Infertile	30 (14, 16)	999	2.1047	**0.0600**	**0.0800**
Jaccard	Fertile	Infertile	30 (14, 16)	999	1.2892	**0.0570**	**0.0725**
Genital, male							
Unweighted UniFrac	Fertile	Infertile	28 (16, 12)	999	2.0719	**0.0180**	**0.0252**
Weighted UniFrac	Fertile	Infertile	28 (16, 12)	999	2.1974	**0.0720**	**0.0877**
Bray–Curtis	Fertile	Infertile	28 (16, 12)	999	1.3802	0.1540	0.1875
Jaccard	Fertile	Infertile	28 (16, 12)	999	1.4820	**0.0060**	**0.0080**
Gut, female							
Unweighted UniFrac	Fertile	Infertile	22 (9, 13)	999	1.0265	0.4250	0.4250
Weighted UniFrac	Fertile	Infertile	22 (9, 13)	999	1.6821	**0.0650**	**0.0827**
Bray–Curtis	Fertile	Infertile	22 (9, 13)	999	1.2738	0.1220	0.1553
Jaccard	Fertile	Infertile	22 (9, 13)	999	1.0695	0.2730	0.3058
Gut, male							
Unweighted UniFrac	Fertile	Infertile	26 (11, 15)	999	1.0341	0.3630	0.3775
Weighted UniFrac	Fertile	Infertile	26 (11, 15)	999	1.4239	0.1470	0.1646
Bray–Curtis	Fertile	Infertile	26 (11, 15)	999	0.9728	0.4960	0.4960
Jaccard	Fertile	Infertile	26 (11, 15)	999	1.0253	0.3650	0.3931

**Table 5:** Differences in the alpha-diversity of EC metagenome between samples. (<0.1 in bold).

Method	Group 1	Group 2	*H*	*p*-Value	*Q*-Value
Genital, female					
Observed	Fertile (14)	Infertile (16)	0.043203	0.835344	0.899601
Shannon	Fertile (14)	Infertile (16)	0.914171	0.33901	0.365088
Evenness	Fertile (14)	Infertile (16)	0.762097	0.382673	0.487039
Genital, male					
Observed	Fertile (16)	Infertile (12)	1.876052	0.170784	0.298273
Shannon	Fertile (16)	Infertile (12)	0.422414	0.515735	0.534836
Evenness	Fertile (16)	Infertile (12)	4.965517	**0.025858**	**0.042589**
Gut, female					
Observed	Fertile (9)	Infertile (13)	1.214047	0.270532	0.360837
Shannon	Fertile (9)	Infertile (13)	4.148272	**0.041677**	**0.055569**
Evenness	Fertile (9)	Infertile (13)	0.491639	0.483197	0.520366
Gut, male					
Observed	Fertile (11)	Infertile (15)	0.29697	0.585788	0.656083
Shannon	Fertile (11)	Infertile (15)	0.032997	0.855858	0.855858
Evenness	Fertile (11)	Infertile (15)	0.56633	0.451721	0.520366

**Table 6:** Differences in the alpha-diversity of KO metagenome between samples. (<0.1 are in bold).

Method	Group 1	Group 2	*H*	*p*-Value	*Q*-Value
Genital, female					
Observed	Fertile (14)	Infertile (16)	0.02765	0.867935	0.914146
Shannon	Fertile (14)	Infertile (16)	0.110599	0.739463	0.739463
Evenness	Fertile (14)	Infertile (16)	1.168203	0.279771	0.340591
Genital, male					
Observed	Fertile (16)	Infertile (12)	2.793103	**0.094671**	0.176719
Shannon	Fertile (16)	Infertile (12)	0.215517	0.642477	0.666272
Evenness	Fertile (16)	Infertile (12)	4.965517	**0.025858**	**0.045251**
Gut, female					
Observed	Fertile (9)	Infertile (13)	0.93757	0.332904	0.490595
Shannon	Fertile (9)	Infertile (13)	5.619844	**0.017758**	**0.023678**
Evenness	Fertile (9)	Infertile (13)	1.695652	0.192858	0.245455
Gut, male					
Observed	Fertile (11)	Infertile (15)	0.420875	0.516501	0.628783
Shannon	Fertile (11)	Infertile (15)	1.245118	0.264487	0.296225
Evenness	Fertile (11)	Infertile (15)	2.505724	0.113433	0.158807

**Table 7:** Differences in the alpha-diversity of MetaCyc pathways between samples. (<0.1 are in bold).

Method	Group 1	Group 2	*H*	*p*-Value	*Q*-Value
Genital, female					
Observed	Fertile (14)	Infertile (16)	0.043212	0.835326	0.866264
Shannon	Fertile (14)	Infertile (16)	0.062212	0.803033	0.803033
Evenness	Fertile (14)	Infertile (16)	0.292051	0.588909	0.610721
Genital, male					
Observed	Fertile (16)	Infertile (12)	3.627809	**0.056822**	**0.093588**
Shannon	Fertile (16)	Infertile (12)	0.105603	0.745206	0.772806
Evenness	Fertile (16)	Infertile (12)	3.448276	**0.063318**	**0.084424**
Gut, female					
Observed	Fertile (9)	Infertile (13)	0.188619	0.664069	0.774747
Shannon	Fertile (9)	Infertile (13)	5.307692	**0.021231**	**0.028309**
Evenness	Fertile (9)	Infertile (13)	2.061315	0.15108	0.183923
Gut, male					
Observed	Fertile (11)	Infertile (15)	0.016881	0.896624	0.896624
Shannon	Fertile (11)	Infertile (15)	0.356229	0.550608	0.616681
Evenness	Fertile (11)	Infertile (15)	1.891582	0.169024	0.197194

**Table 8:** Differences in the beta-diversity of EC metagenome between samples. (PERMANOVA, <0.1 are in bold).

Method	Group 1	Group 2	Sample Size	Permutations	Pseudo-F	*p*-Value	*Q*-Value
Genital, female							
Bray–Curtis	Fertile	Infertile	30 (14, 16)	999	2.92481	**0.061**	**0.074261**
Jaccard	Fertile	Infertile	30 (14, 16)	999	0.939749	0.389	0.473565
Genital, male							
Bray–Curtis	Fertile	Infertile	28 (16, 12)	999	1.82435	0.121	0.141167
Jaccard	Fertile	Infertile	28 (16, 12)	999	1.941499	**0.08**	0.106667
Gut, female							
Bray–Curtis	Fertile	Infertile	22 (9, 13)	999	1.985473	**0.019**	**0.025333**
Jaccard	Fertile	Infertile	22 (9, 13)	999	0.961617	0.462	0.497538
Gut, male							
Bray–Curtis	Fertile	Infertile	26 (11, 15)	999	1.30249	0.184	0.190815
Jaccard	Fertile	Infertile	26 (11, 15)	999	0.813824	0.632	0.655407

**Table 9:** Differences in the beta-diversity of KO metagenome between samples. (PERMANOVA, <0.1 are in bold).

Method	Group 1	Group 2	Sample size	Permutations	Pseudo-F	*p*-Value	*Q*-Value
Genital, female							
Bray–Curtis	Fertile	Infertile	30 (14, 16)	999	2.836859	**0.048**	**0.061091**
Jaccard	Fertile	Infertile	30 (14, 16)	999	0.940447	0.42	0.511304
Genital, male							
Bray–Curtis	Fertile	Infertile	28 (16, 12)	999	1.689142	0.146	0.157231
Jaccard	Fertile	Infertile	28 (16, 12)	999	2.134797	**0.065**	**0.086667**
Gut, female							
Bray–Curtis	Fertile	Infertile	22 (9, 13)	999	1.930443	**0.034**	**0.045333**
Jaccard	Fertile	Infertile	22 (9, 13)	999	0.90927	0.518	0.604333
Gut, male							
Bray–Curtis	Fertile	Infertile	26 (11, 15)	999	1.441061	0.113	0.137565
Jaccard	Fertile	Infertile	26 (11, 15)	999	0.808572	0.573	0.64176

**Table 10:** Differences in the beta-diversity of MetaCyc pathways between samples. (PERMANOVA, <0.1 are in bold).

Method	Group 1	Group 2	Sample Size	Permutations	Pseudo-F	*p*-Value	*Q*-Value
Genital, female							
Bray–Curtis	Fertile	Infertile	30 (14, 16)	999	1.549936	0.189	0.203538
Jaccard	Fertile	Infertile	30 (14, 16)	999	0.830535	0.477	0.580696
Genital, male							
Bray–Curtis	Fertile	Infertile	28 (16, 12)	999	1.585594	0.155	0.1736
Jaccard	Fertile	Infertile	28 (16, 12)	999	2.071996	**0.09**	0.12
Gut, female							
Bray–Curtis	Fertile	Infertile	22 (9, 13)	999	2.164885	**0.037**	**0.049333**
Jaccard	Fertile	Infertile	22 (9, 13)	999	0.690923	0.599	0.645077
Gut, male							
Bray–Curtis	Fertile	Infertile	26 (11, 15)	999	1.106805	0.303	0.314222
Jaccard	Fertile	Infertile	26 (11, 15)	999	0.511691	0.781	0.809926

**Table 11:** Differentially abundant metagenome features in gut and genital samples.

	Body Site	Class	Biomarker	Function
EC #	Genital	Infertile	EC 6.3.5.5	Ligases; forming carbon nitrogen bonds.
			EC 2.7.8.20	Transferases; transferring phosphorous containing group
			EC 5.1.1.13	Isomerases; acting on amino acids and derivatives.
			EC 3.1.2.21	Hydrolases; acting on ester bonds
			EC 3.4.14.11	Hydrolases; acting on peptide bonds (peptidases)
			EC 2.4.1.8	Transferases; glycosyltransferases; hexosyltransferases
			EC 2.4.2.6	Nucleoside deoxyribosytransferase; catalyses the cleavage of the glycosidic bonds of 2-deoxyribonucleosides.
			EC 3.2.1.70	Hydrolases; glycosidases, i.e., enzyme that hydrolyse O- and S-glycosyl compounds.
			EC 2.7.1.76	Transferases; transferring phosphorous containing group
			EC 1.1.3.21	Oxidoreductase; acting on the CH-OH group of donors; with oxygen as acceptors.
			EC 2.7.7.61	Transferases; transferring phosphorous containing group
			EC 6.3.4.4	Ligases; forming carbon nitrogen bonds.
		Fertile	EC 1.8.4.12	Oxidoreductase; acting on a sulphur group of donors; with a disulphide as acceptor
			EC 1.3.8.6	Oxidoreductase; acting on the CH-CH group of donors; with flavin as acceptor.
	Gut	Infertile	EC 6.2.1.5	Ligases; forming carbon sulphur bonds.
		Fertile	EC 3.1.3.18	Hydrolases; acting on ester bonds
KEGG Ortholog ID	Gut	Infertile	K00057: Glycerol-3-phosphate dehydrogenase	Pathways: Glycerophospholipid metabolism; Biosynthesis of secondary metabolite
	Genital		K00041: Tagaturonate reductase	Pathways: pentose and glucoronate interconversions; metabolic pathways.
MetaCyc Pathway ID	Gut	Infertile	KDO-NAGLIPASYN-PWY	Pathway: Superpathway of (Kdo)_2_-lipid A biosynthesis
			PWY-6519	Pathway: 8-amino-7-oxononanoate biosynthesis 1
			PWY-5861	Pathway: Superpathway of demethylmenaquinol-8 biosynthesis 1
			BIOTIN-BIOSYNTHESIS-PWY	Pathway: Biotin biosynthesis 1
			PWY0-1479	Pathway: t-RNA processing
			PWY-5838	Pathway: Superpathway of menaquinol-8 biosynthesis 1
			PWY0-845	Pathway: Superpathway of pyridoxal 5′-phosphate biosynthesis and salvage
			PYRIDOXSYN-PWY	Pathway: pyridoxal 5′-phosphate biosynthesis 1
		Fertile	PWY-7221	Pathway: guanosine ribonucleotides de novo biosynthesis.

## Data Availability

The data presented in this study are available on request from the authors and after appropriate IRB approvals.
